# Intraspecific diversity among partners drives functional variation in coral symbioses

**DOI:** 10.1038/srep15667

**Published:** 2015-10-26

**Authors:** John Everett Parkinson, Anastazia T. Banaszak, Naomi S. Altman, Todd C. LaJeunesse, Iliana B. Baums

**Affiliations:** 1 Department of Biology, The Pennsylvania State University, University Park, PA 16802, USA; 2 Unidad Académica de Sistemas Arrecifales, Instituto de Ciencias del Mar y Limnología, Universidad Nacional Autónoma de México, Puerto Morelos, Q. Roo 77580, México; 3 Department of Statistics, The Pennsylvania State University, University Park, PA, 16802, USA

## Abstract

The capacity of coral-dinoflagellate mutualisms to adapt to a changing climate relies in part on standing variation in host and symbiont populations, but rarely have the interactions between symbiotic partners been considered at the level of individuals. Here, we tested the importance of inter-individual variation with respect to the physiology of coral holobionts. We identified six genetically distinct *Acropora palmata* coral colonies that all shared the same isoclonal *Symbiodinium ‘fitti’* dinoflagellate strain. No other *Symbiodinium* could be detected in host tissues. We exposed fragments of each colony to extreme cold and found that the stress-induced change in symbiont photochemical efficiency varied up to 3.6-fold depending on host genetic background. The *S. ‘fitti’* strain was least stressed when associating with hosts that significantly altered the expression of 184 genes under cold shock; it was most stressed in hosts that only adjusted 14 genes. Key expression differences among hosts were related to redox signaling and iron availability pathways. Fine-scale interactions among unique host colonies and symbiont strains provide an underappreciated source of raw material for natural selection in coral symbioses.

Reef ecosystems thrive thanks to a mutualism between scleractinian corals and photosynthetic dinoflagellates in the genus *Symbiodinium*^1^. These endosymbiotic algae are sheltered within host cells, so biochemical changes in one partner directly impact the other's cellular environment^2^. Sustained hot or cold stress can cause symbiont loss known as coral bleaching^3^,^4^, driving fitness consequences ranging from reduced host reproductive output to colony death^5^. These selective pressures act on physiological variation among coral-dinoflagellate symbioses, potentially leading to local adaptation of each partner^6^,^7^. However, the contribution of physiological variation among individuals to functional variation among combined holobionts is poorly understood.

Phenotypic differences among individuals engaged in symbiosis act synergistically to expand the range of holobiont functional diversity subject to selective pressures^8^. Such interactive genetic effects are observed in diverse systems including insect-bacteria, plant-bacteria, and plant-fungus associations^9^. Climate change intensifies selection among coral holobionts, but uncertainty surrounds the rate at which corals and their symbionts may acclimate or adapt to a changing environment^10^. Adjacent colonies with identical host and symbiont compositions at the species level show different bleaching susceptibilities^11^,^12^, indicating that intraspecific variation exists. Despite renewed emphasis on the fundamental concept that natural selection acts on variation among individuals within species^13^, our understanding of the adaptive significance of fine-scale genetic effects in coral holobionts is still in its infancy^9^.

To date, most studies have focused on stress performance among corals associating with unique *Symbiodinium* taxa (ranging from species to higher order clades). For a given coral species, colonies paired with stress-resistant symbiont taxa often perform differently than colonies paired with stress-sensitive taxa. Such colonies might be more tolerant to increased temperatures^14^, grow more slowly^15^, exhibit altered transcription^16^, and possess distinct heritabilities for holobiont traits^17^. Interactions below the species level have received less attention, though they may produce similar effects^18^. For example, at the population level, holobiont thermotolerance and host gene expression vary between two genetically-differentiated populations of a coral species despite sharing one symbiont species across sites^19^,^20^. Conversely, thermotolerance varies among groups of coral juveniles reared with different populations of a particular symbiont species despite belonging to a single host population^7^.

Differences have also been observed among individuals within coral populations. Experimental crosses between genotyped colonies reveal incompatibilities among parents in terms of fertilization rates and larval survival^21^. Moreover, batches of symbiont-free, half-sibling larvae exhibit different physiological and molecular responses to temperature stress depending on the identity of the sperm donor^21^,^22^. However, host genotype effects on symbiont performance have not been documented in adult colonies because the symbiont community has not been measured at the same fine-scale resolution. Although manipulation of the symbiont community is possible in coral juveniles, a major drawback of this approach is that during early development most corals lack the *Symbiodinium* specificity characteristic of adults^23^. Thus, juvenile symbiosis dynamics are not necessarily representative of mature associations.

Here, we investigated functional diversity among coral holobionts at the finest scale possible, using neutral markers to resolve both partners to the level of individuals within species. We took advantage of the *Acropora palmata—Symbiodinium ‘fitti’* system, where the mature coral usually associates with just one symbiont species and each host colony harbors only one asexually-derived symbiont strain^24^. This allowed us to identify genetically distinct host colonies sharing the same *S. ‘fitti’* strain. We exposed these colonies to cold temperature shock to test the hypothesis that host genotype influences the photochemical stress response of a resident clonal symbiont *in hospite*. We also tracked host gene expression to identify molecular pathways involved in the interaction.

## Results

### Host diversity and symbiont uniformity

Based on microsatellite alleles, there were at least 15 *Acropora palmata* genotypes and nine *Symbiodinium ‘fitti’* strains present on the reef sampled in Puerto Morelos, Mexico (Supplementary Table S1). In the experimental colonies (highlighted in Supplementary Table S1 and referred to as host multilocus genotypes *A*, *B*, *D*, *X*, *Y*, and *Z*), only one *Symbiodinium* ITS2 type was present in each colony as indicated by the ITS2 DGGE profile and sequence characteristic of *S. ‘fitti’* (*sensu Symbiodinium* type A3 from the Caribbean, Genbank Accession: AF333507)^25^. More sensitive qPCR assays failed to detect any background symbionts from the other clades known to associate with Caribbean corals (Clades B, C, and D; Supplementary Fig. S1). Furthermore, each colony contained only a single strain (clone) of *S. ‘fitti’* based on allele homogeneity at all loci. Thus, each holobiont represented a unique pairing of a single host and a single symbiont genotype, where all hosts were distinct and all resident symbiont populations were essentially identical. All six colonies were located in close proximity to each other. Fragments of each colony were exposed to cold shock in aquaria.

### Symbiont photochemistry

**Figure 1:**
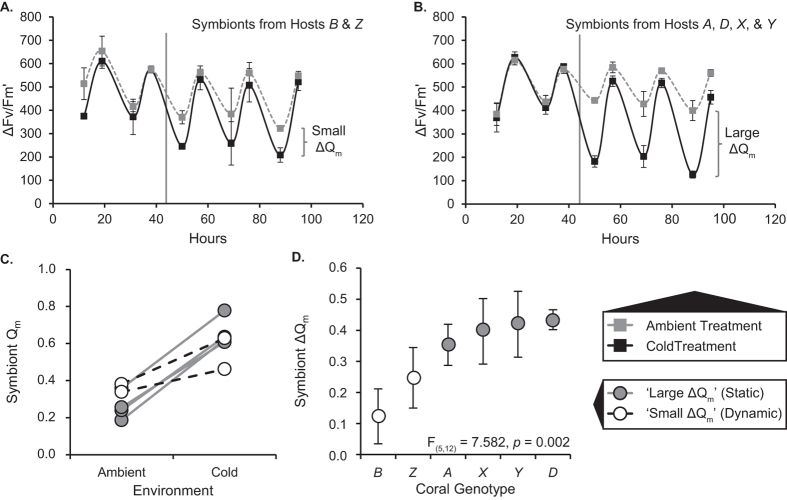
Physiology of an isoclonal *Symbiodinium ‘fitti’* strain found in six genetically distinct *Acropora palmata* colonies. Shown are diurnal oscillations in the quantum yield of charge separation for photosystem II over the course of the experiment for (**A**) ‘small ΔQ_m_’ colonies (hosts *B* and *Z*; later referred to as ‘Dynamic’) and **(B)** ‘large ΔQ_m_’ colonies (hosts *A, D, X,* and *Y*; later referred to as ‘Static’). The vertical line indicates when cold shock treatment began. Error bars represent 95% confidence intervals for either two or four holobiont measurements, respectively. **(C)** Reaction norm of pressure over photosystem II (Q_m_) for ambient and cold exposure. **(D)** The difference in pressure over photosystem II between cold and ambient exposure (ΔQ_m_). Error bars represent 95% confidence intervals for three measurements per fragment (one per day of exposure).

The photochemistry of the *S. ‘fitti’* strain as measured by the quantum yield of charge separation of photosystem II (ΔF/F_m_?; Fig. 1A,B) and the maximum excitation pressure over photosystem II (Q_m_; Fig. 1C) varied under ambient (27 °C) and cold (20 °C) conditions. Although solar radiation and Q_m_ fluctuated from day to day, the cold treatment effect (ΔQ_m_) remained remarkably stable (Fig. 1A,B). A repeated measures analysis revealed that the day of observation was not a significant factor (ANOVA, *F*_2,10_ = 0.738, α = 0.05, *p* = 0.502); we therefore treated values from different days as technical replicates to obtain measures of error for each host background. All data sets were normally distributed.

Holobionts with clonal symbionts differed in ΔQ_m_ (ANOVA, *F*_5,12_ = 7.582, α = 0.05, *p* = 0.002; Fig. 1D), with the average change under cold stress ranging from +0.12 ± 0.08 (s.d.) in the host *B* background to +0.43 ± 0.03 (s.d.) in the host *D* background. Positive values indicate a decrease in photochemical efficiency, so while all holobionts were negatively impacted by cold shock, the effect on the *S. ‘fitti’* strain was less pronounced in certain host backgrounds (those with ‘small ΔQ_m_’) than in others (those with ‘large ΔQ_m_’). A post-hoc test confirmed the existence of two homogenous subgroups: the ‘small ΔQ_m_’ group containing colonies *B* and *Z*, and the ‘large ΔQ_m_’ group containing colonies *Z*, *A*, *D*, *X*, and *Y* (Tukey's HSD, α = 0.05). Although colony *Z* was statistically intermediate, we elected to classify it in the ‘small ΔQ_m_’ group on the basis that all of its ΔQ_m_ values (3/3) fell below the mean for all ΔQ_m_ observations. This made it more similar to colony *B* (3/3 below the mean) than to colonies *A* (1/3 below the mean)*, D* (0/3 below the mean)*, X* (1/3 below the mean), and *Y* (0/3 below the mean).

When testing for other differences among holobionts prior to the experimental treatment, we observed deviations in symbiont density (ANOVA, *F*_5,24_ = 76.34, α = 0.05, *p* < 0.001; Supplementary Fig. S2A) but not cell ellipsoid volume (ANOVA, *F*_5,234_  = 1.78, α = 0.05, *p* = 0.119; Supplementary Fig. S2C). ΔQ_m_ did not correlate with either metric in simple linear regressions (density: R^2^ = 7.4%, n = 6, α = 0.05, *p* = 0.603; volume: R^2^ = 8.1%, n = 6, α = 0.05, *p* = 0.536; Supplementary Fig. S2B,D).

### Host gene expression

**Figure 2:**
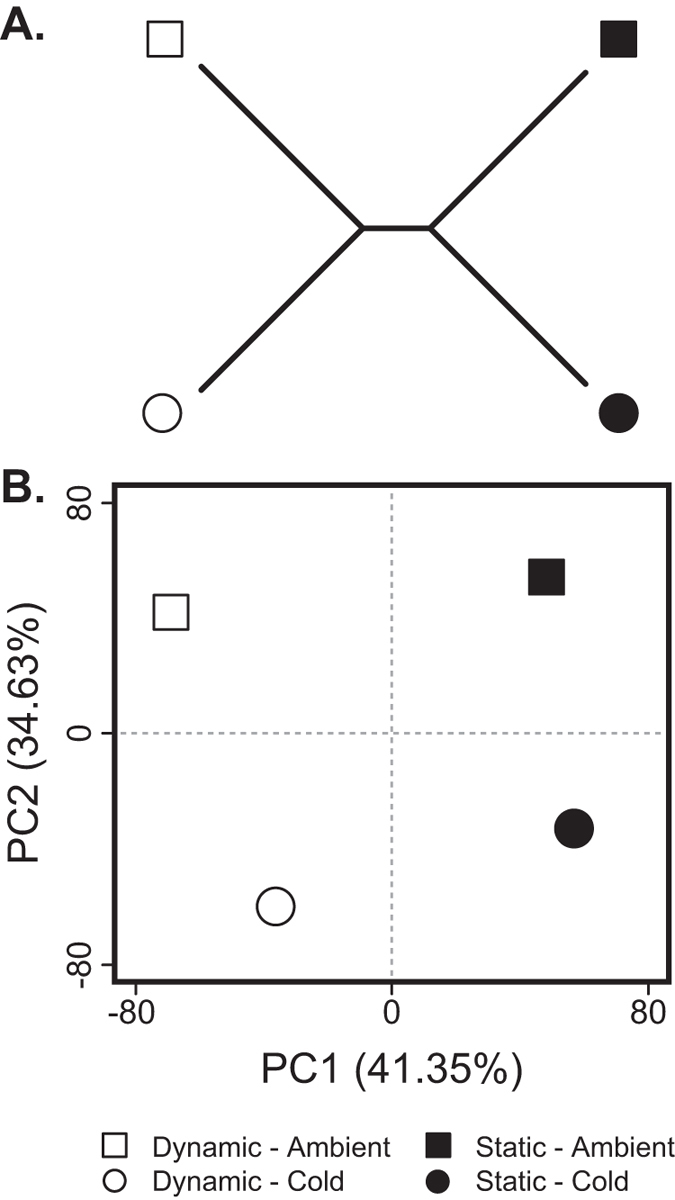
*Acropora palmata* microarray clustering. **(A)** Unrooted hierarchical clustering of samples based on expression profiles as calculated in R using the hclust function and complete linkage agglomeration method. **(B)** Principle components 1 and 2 (x- and y-axis, respectively) of sample gene expression as calculated in R using the prcomp function and a covariance matrix. PC1 is associated with colony phenotype (Static or Dynamic), while PC2 is associated with temperature treatment (cold or ambient).

Using a host-specific microarray to contrast the most extreme colonies (*B* and *Z* vs. *D* and *Y*), the combination of two photochemical phenotypes and two temperature treatments yielded four gene expression profiles. Hierarchical clustering and principle component analysis (PCA) on expression profiles supported similar groupings (Fig. 2A,B). The first PCA axis explained a majority of the total variation (41.35%) and corresponded to a split between ‘small ΔQ_m_’ (a.k.a. Dynamic) colonies and ‘large ΔQ_m_’ (a.k.a. Static) colonies (see below). The second PCA axis explained 34.63% of the total variation and corresponded to a split between cold and ambient temperature treatments.

**Figure 3:**
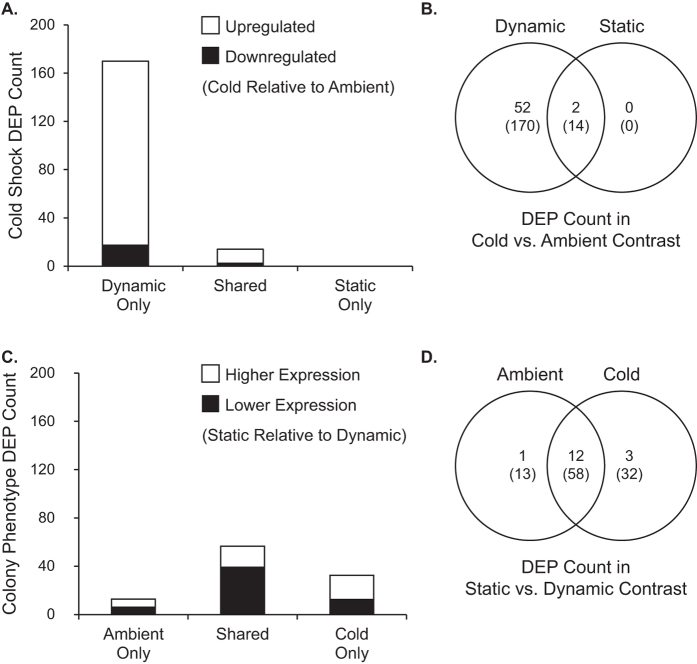
Differential expression quantification. **(A)** The total number of differentially expressed probes (DEPs) in response to temperature (‘cold’ or ‘ambient’) that were unique or shared among Static or Dynamic hosts and **(B)** overlap in the number of annotated, nonredundant, differentially expressed genes (top number) and probes (bottom number in parentheses) that were significant (FDR *q* < 0.05) for cold vs. ambient contrasts. **(C)** The total number of probes differentially expressed in response to colony phenotype (‘large’ or ‘small’ ΔQ_m_; aka ‘Static’ or ‘Dynamic’) that were unique or shared among cold- or ambient-treated hosts and **(D)** overlap in the number of annotated, nonredundant, differentially expressed genes (top number) and probes (bottom number in parentheses) that were significant (FDR *q* < 0.05) for Static vs. Dynamic colony phenotype contrasts.

**Figure 4:**
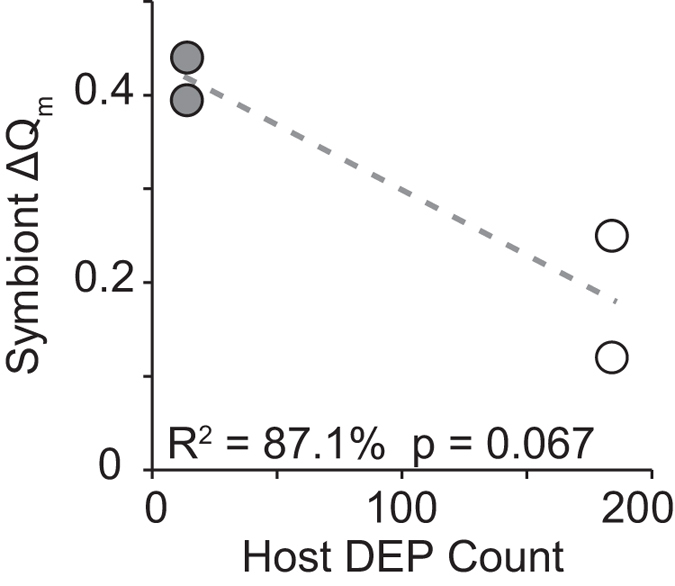
Correlation between the photochemistry (ΔQ_m_) of an isoclonal *Symbiodinium ‘fitti’* strain and gene expression of four *Acropora palmata* genotypes (number of differentially expressed probes, or DEPs). White fills indicate Dynamic host gene expression phenotypes, while gray fills represent Static host gene expression phenotypes. Note that combining replicates for the differential expression analysis led to dependence among DEP counts within photochemistry categories.

Under cold shock, hosts with symbionts showing a ‘small ΔQ_m_’ photochemical response significantly altered expression of 184 probes (FDR *q* < 0.05; Fig. 3A,B). These differentially expressed probes (DEPs) comprised 54 unique genes with annotation information (Supplementary Table S2). Given the relatively large transcriptional response, we refer to these holobionts as ‘Dynamic.’ In contrast, hosts with symbionts showing a ‘large ΔQ_m_’ response altered only 14 DEPs comprising 2 unique, annotated genes (Fig. 3A,B; Supplementary Table S2). We refer to these holobionts as ‘Static.’ Within temperature treatments, there was a total of 103 DEPs in the Dynamic vs. Static holobiont contrast—16 unique, annotated genes—indicating fixed expression differences (Fig. 3C,D; Supplementary Table S3). No transcripts showed a photochemical phenotype by temperature treatment interaction. ΔQ_m_ and total number of cold shock DEPs were marginally correlated at a relaxed α = 0.1 (R^2^ = 87.1%, *p* = 0.067; Fig. 4), but note the small sample size (n = 4) and the dependence among DEP counts within photochemistry categories due to combining replicates for the differential expression analysis.

**Figure 5:**
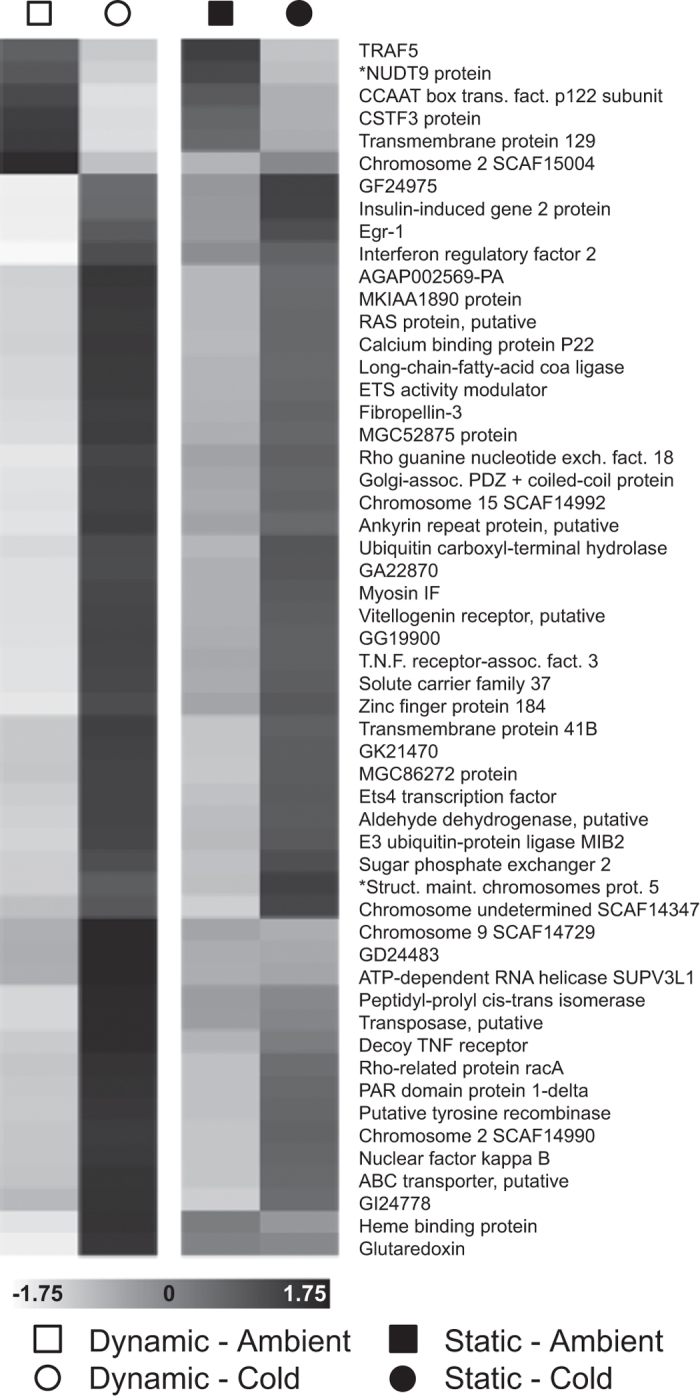
Expression heatmaps. The transcription profile for all 54 annotated genes with significant differential expression in Dynamic hosts responding to cold stress (FDR *q* < 0.05) is shown as a heatmap of scaled expression coefficients (standard scores above or below the gene mean), along with a heatmap for the same genes in Static hosts. Asterisks precede genes that also showed significant expression differences in Static hosts (FDR *q* < 0.05).

When comparing cold stress responses in each group side-by-side (Fig. 5), many of the significantly upregulated genes in Dynamic hosts were also upregulated in Static hosts, but the magnitude of the change was small, rendering them statistically insignificant. Genes differentially expressed between cold and ambient temperatures in Dynamic holobionts were involved in redox maintenance, transmembrane transport, and calcium and redox signaling. The gene list was functionally enriched for the GO terms signal transduction, response to stimulus, (ribo)nucleotide metabolic processes, and biological regulation. Static hosts were fixed for greater expression of genes involved in oxidative stress response (specifically iron availability) and redox signaling regardless of temperature treatment. This gene list was functionally enriched for ion binding.

## Discussion

We found that a single, isoclonal *Symbiodinium ‘fitti’* strain’s photochemical response to cold shock varied when engaged in symbiosis with six genetically distinct *Acropora palmata* colonies (Fig. 1). These responses correlated with detectable host transcription changes (Figs 2, 3, 4, 5). Differences among colonies could not be attributed to background symbiont strains (Supplementary Fig. S1), symbiont cell density (Supplementary Fig. S2A,B), symbiont size (Supplementary Fig. S2C,D), or environmental heterogeneity. Because the dominant symbiont cells were genetically uniform at ten microsatellite markers, and no other *Symbiodinium* were detected, host genotype emerged as the likely factor determining symbiont stress response phenotype in our experiment.

Genetic variation among *S. ‘fitti’* strains as identified with 10 microsatellite loci is far greater than variation within strains^24^. However, even small somatic differences can result in phenotypic differences between ‘clonal populations’ in some systems (*e.g. Cryptococcus neoformans*)^26^. Assessing intracolony heterogeneity in the performance of the dominant symbiont strain was outside the scope of this work, but such diversity, if present, might have also contributed to the observed variation in ΔQ_m_ along with host genotype. Additional factors that may have been important but could not be accounted for in this experiment included the impact of other organisms associated with the coral microbiota (*e.g.* archae, viruses, and bacteria), environmental microvariation at scales <0.5 m, and epigenetic factors. However, epigenetic factors would be confounded with host genotype, and environmental microvariation was at least absent in the aquaria. If members of the microbial community other than *Symbiodinium* played a role, they would have had to differ consistently among host genotype groups. Hence, while these factors represent interesting targets for future studies, the observed differences in symbiont stress response are difficult to explain without considering the host genotype as important.

Cold temperatures slow the rates of electron transport and carbon fixation, decreasing photosystem II yield (ΔF/F_m_?) and increasing maximal excitation pressure (Q_m_). As expected, we observed elevated Q_m_ in all six holobiont fragments exposed to cold shock. However, the magnitude of change in pressure (ΔQ_m_) varied up to 3.6-fold among holobionts despite the clonality of the *S. ‘fitti’* strain shared by all colonies. Multiple host expression changes took place in colonies where the *S. ‘fitti’* photoresponse was minimal (‘Dynamic’ hosts), whereas in the remaining colonies, host transcription changes were muted (‘Static’ hosts) and symbionts experienced greater fluctuation and diminishment in photochemical efficiency (Fig. 4). These responses may indicate a phenotypic buffering effect^27^, where symbiont performance is maintained within a narrow physiological range when occurring within Dynamic hosts.

Based on gene identities and expression patterns, we propose that the capacity of the host to manage its cellular environment, specifically redox state and iron availability, affects the resident symbiont’s cold stress response. We observed three types of gene expression responses: (1) cold shock genes that changed in Dynamic hosts but not Static hosts, (2) cold shock genes that changed in both types of host (but to a more extreme degree in Dynamic hosts), and (3) fixed differences in gene expression between Static and Dynamic hosts. We discuss examples of each expression pattern below.

**Figure 6:**
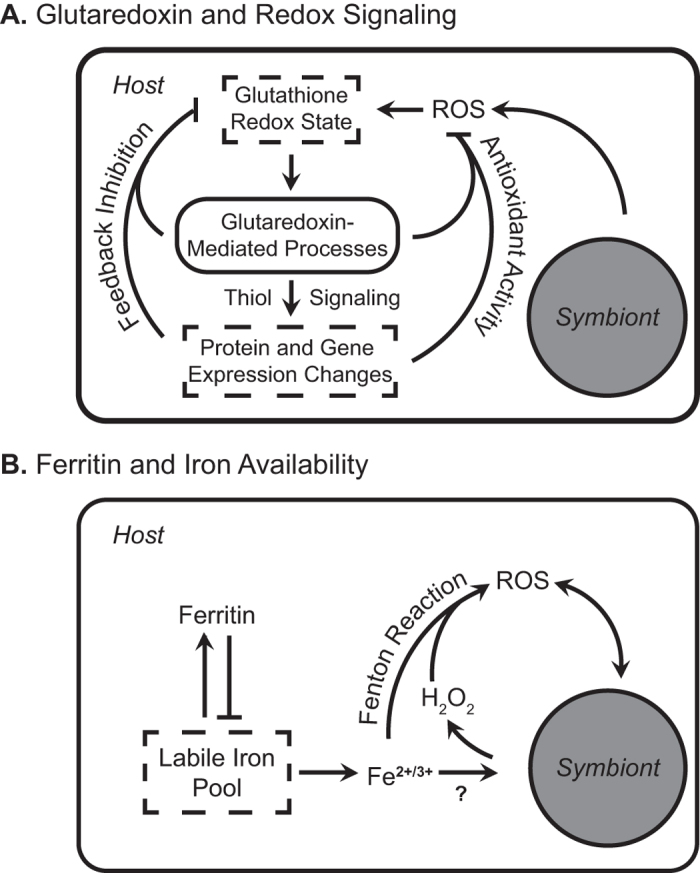
Mechanistic models for molecular interactions between partners related to (A) glutaredoxin and redox signaling and (B) ferritin and iron availability. Lines connect interacting molecules or processes. Terminal arrows indicate activation or enhancement, terminal straight lines indicate inhibition. Terms in dashed squares represent targets of positive or negative regulation.

The first category of genes includes those that changed only in Dynamic hosts under thermal stress. For example, Dynamic corals increased glutaredoxin expression 5.6-fold in response to cold shock, while expression remained constant in Static corals. Glutaredoxins facilitate electron transfer in the glutathione cycle, which helps to maintain cellular redox homeostasis (Fig. 6A)^28^. Cold shock inhibits photochemistry and induces *Symbiodinium* to generate reactive oxygen species (ROS) such as hydrogen peroxide (H_2_O_2_), which can diffuse into host cells^29^. Because H_2_O_2_ can serve as an electron carrier in the glutathione cycle^30^, stressed symbionts may directly disrupt host redox homeostasis, signaling the host nucleus much like a plant chloroplast signals the plant nucleus during stress^31^. In turn, the host may modify its cellular environment through glutaredoxin-mediated thiol signaling, which can induce downstream transcriptional adjustments that include antioxidant activity^28^. Such adjustments may reduce the stress experienced by the symbiont, perhaps explaining why *Symbiodinium* in Dynamic hosts appeared to be less compromised during cold shock.

The second category includes a set of cold shock genes that were upregulated in all corals, but the magnitude of the change was smaller in Static hosts relative to Dynamic hosts. As a result, only the changes in Dynamic hosts were statistically significant. This pattern suggests that different coral genotypes fundamentally respond to cold stress in similar ways. Rather than using a separate set of genes that may have buffered symbiont performance, Dynamic hosts accentuated an existing response. The mechanism may relate once again to glutaredoxin. For example, eight ‘accentuated’ genes encoded proteins with disulfide bonds (Supplementary Table S3), which can be targeted by glutaredoxin-mediated thiol signaling^32^. Because Static hosts did not adjust glutaredoxin levels, they may not have been able to mount a strong transcriptional response (Fig. 5). Regardless of the mechanisms involved, stress-related expression polymorphisms that vary by degree rather than identity are evident within coral populations^33^, and here we show that at least some of them correspond to variation in the performance of a *Symbiodinium* clone.

The third and final category includes genes that showed fixed differences in expression among coral genotypes regardless of the treatment temperature. For hosts with Static gene expression patterns, both control and experimental colonies appeared to exhibit greater levels of sustained expression in certain genes relative to that found in hosts with Dynamic expression patterns. For example, three ferritin-related genes were expressed 5- to 22-fold higher in Static hosts under both ambient and cold conditions (Supplementary Table S3). Ferritin acts as an iron-sequestration molecule, importantly reducing the availability of free iron for spontaneous ROS generation via the Fenton pathway (Fig. 6B)^34^. *Symbiodinium* appear to be iron-limited *in hospite*^35^, and likely rely on the host as an iron source. Because ferritin expression levels generally reflect the size of the free iron pool in the cell^36^, host ferritin levels may dictate symbiont iron limitation, affecting both symbiont and host performance. This may explain why at least one ferritin gene in *A. palmata* appears to be under intense positive selection^37^.

The maintenance of phenotypic polymorphisms may be favored in the presence of spatial and temporal environmental variability^38^. We observed distinct stress responses among the six holobionts in our study; had more genotypes been included in the experiment, more extreme and intermediate phenotypes might have been recovered. While each phenotype is apparently functional, relative success may depend on whether the environment is stable or fluctuating. For example, Static hosts with elevated ferritin (and therefore high levels of free iron available to their symbionts) may benefit from enhanced *Symbiodinium* performance provided conditions remain stress-free. Indeed, we found improved photochemical efficiency (*i.e.* lower ambient Q_m_) among Static colonies at ambient temperature relative to Dynamic colonies (Fig. 1C; t-test, *p* = 0.049). During temperature anomalies, however, excess iron ions may become a liability as they promote ROS production and tissue damage^39^,^40^. In contrast, the symbionts in Dynamic hosts are not as photochemically efficient at ambient temperature, but their efficiency does not decline as severely during stress. Such a trade-off may support polymorphism within the mutualism. Further experiments encompassing more genotypes, more natural temperature conditions, and longer acclimation periods will enable a better understanding of the ecological relevance of these findings.

Hot and cold temperature anomalies can lead to widespread coral mortality^12^,^41^, guiding the outcomes of natural selection^42^. Given that physiological variation exists within species of coral hosts and dinoflagellate symbionts, it is important to recognize that the holobiont is a unit of selection in these symbioses^12^,^43^,^44^,^45^. The variation resulting from interactions between particular host and symbiont genotypes may play a role in the evolutionary response to climate change, an important consideration when predicting the status of coral mutualisms in the future^46^. Selection may be particularly strong for alleles affecting the molecular pathways linked to symbiosis maintenance and photochemistry during temperature stress, such as those outlined here. Intriguingly, only Dynamic hosts appeared to participate in the annual synchronized spawning event in Puerto Morelos, whereas none of the Static hosts spawned (Parkinson *et al.*, personal observation). Though anecdotal, this pattern is compelling because it suggests holobionts composed of different host-symbiont genotype pairings may also have different fitness outcomes, providing raw material for natural selection.

This study provides new evidence of functional diversity among individual coral colonies. Our finding that genotype interactions among host and symbiont individuals can influence population-level dynamics emphasizes the need to preserve existing genotypic richness in coral populations^18^. While not investigated here, the maintenance of symbiont genotypic diversity may also be important when managing reefs or designing coral restoration nurseries. Though it is rare to find adult *A. palmata* coral clone mates with different *S. ‘fitti’* strains^24^, such cases will be necessary to test how holobiont physiology varies with symbiont genotype, to provide a more concrete link between genotype interactions and fitness effects, and to draw accurate conclusions about micro-coevolution. Coral-dinoflagellate interactions have rarely been studied at this level of genetic resolution in the past, but given their potential ecological and evolutionary significance, they merit further investigation.

## Materials and Methods

### Study system

The Caribbean Elkhorn coral, *Acropora palmata*, primarily associates with one phylogenetic lineage of *Symbiodinium* (ITS2 type A3)^47^. Based on hierarchical molecular markers (LaJeunesse *et al.* unpublished data), the Caribbean A3 lineage represents a cohesive species, and is provisionally termed *Symbiodinium ‘fitti*’ *nomen nudum*^48^. The host spawns symbiont-free gametes, so *S. ‘fitti’* cells must be acquired from the environment by each generation. Within host tissues, the haploid symbiont mostly propagates asexually via cell division^24^,^49^, meaning each coral can be viewed as a culture vessel for a single symbiont strain.

### Host and symbiont genotyping

In Spring 2011, colonies of *A. palmata* (n = 20) were sampled from La Bocana Chica Reef in the Puerto Morelos Reef National Park, Mexico (N 20°52.461′, W 86°51.073′). For each colony, the host was genotyped at five neutral microsatellite loci^50^, and the *S. ‘fitti’* strains were genotyped at ten microsatellite loci^48^. These ten markers were sensitive enough to detect multiple strains in a single host colony provided the minor strains each represented ≥5% of the symbiont community^24^. Tissues of colonies sharing identical alleles at all *A. palmata* loci (or *S. ‘fitti’* loci) were deemed to be clone mates of the same host genotype (or symbiont strain). The chance of misidentifying two colonies (or strains) as clonal when in fact they were distinct (the probability of identity) was on the order of 10^−7^ for the host (or 10^−5^ for the symbiont)^24^.

Six holobionts (the ‘experimental colonies’) were targeted for further study based on three criteria: each colony hosted only one isoclonal *S. ‘fitti’* strain; the symbiont strain was identical across colonies; and each host represented a unique genotype (see Results). All colonies were found at the same depth (~3 m) distributed between two clusters ~30 m apart: one with four colonies and one with two colonies. Within each cluster, all colonies were located within 0.5 m of each other, and it was later revealed that each cluster included at least one Dynamic coral and one Static coral. Though disturbances can contribute to intraspecific physiological variation^51^, this 'natural common garden' reduced historical environmental heterogeneity. For microhabitat variation to account for the observed differences among colonies, it would have had to occur over spatial scales <0.5 m. All colonies greatly exceeded minimum reproductive size (55 cm along the longest length of live tissue)^52^, and were therefore expected to spawn.

Because corals sometimes host more than one species of *Symbiodinium* from different clades, the experimental colonies were further screened with denaturing gradient gel electrophoresis (DGGE) following PCR of the ITS2 region, which detects sub-cladal types that represent ≥5–10% of the total symbiont community^25^,^47^. The region was amplified using the primers ITSintFor2 and ITS2CLAMP and bands were visualized on a denaturing gradient gel^53^. Representative bands were excised, re-amplified with the same primers less the GC-rich clamp, and directly sequenced on an Applied Biosciences sequencer (Applied Biosciences, Foster City, CA, USA) at the Pennsylvania State University Genomics Core Facility. Electropherograms were checked visually using CodonCode Aligner software (CodonCode, Dedham, MD, USA).

Though background *Symbiodinium* from Clades B, C, and D have occasionally been detected in *A. palmata* at low abundance^54^, only *S. ‘fitti’* strains from Clade A are present at appreciable levels in most colonies throughout the Caribbean^24^,^47^. Nevertheless, we used clade-specific qPCR assays to test for the presence of these other coral-associated Caribbean symbionts (for methodology, see Supplementary Fig. S1)^55^,^56^. We did not check for the primarily free-living, Pacific, or foram/sponge-associated Clades E-I, as their detection would more likely be explained by environmental contamination rather than true endosymbiosis in this system.

### Cold stress experiment

In Summer 2011, single fragments (~30 cm^2^) from the six experimental colonies were collected with hammer and chisel from the growing tip at the top of each coral and transported to a 45 L polycarbonate bin containing filtered seawater maintained at 29 °C to match that day’s reef conditions. The outdoor bin was covered with a neutral density shade cloth that reduced natural irradiance by ~50% relative to full sunlight. The fragments were subdivided into 2 separate pieces of ~10 cm^2^ each containing tens of individual polyps. After 2 d of acclimation, the temperature treatments began. One piece of each colony was transferred to a shaded 45 L polycarbonate bin containing filtered seawater precooled to ambient (27 °C) conditions. The other piece of each colony was transferred to a bin precooled to extreme cold conditions (20 °C). Temperatures in the bins were maintained with aquarium chillers (Current-USA, CA, USA). Water was circulated with an aquarium pump and changed daily with additional precooled filtered seawater. The fragments were maintained in the treatments for 3 d (until the evening of the third day of exposure) to ensure that the photochemical response was stable. HOBO data loggers (Onset Co., MA, USA) in each bin confirmed that temperatures stayed within ±0.4 °C of the target for each treatment.

The ambient temperature was decreased slightly relative to reef temperature to reduce the risk of unintended bleaching during the experiment. The cold temperature was extreme compared to what the colonies would naturally experience on the reef. Temperatures were not ramped, such that the corals were exposed to instantaneous temperature shock. Given time restrictions at the field site and the expectation that intraspecific differences might be subtle, the exposure was designed not to mimic natural conditions, but to accentuate differences in acute cold shock responses among holobionts. Despite the extreme conditions, all corals survived the experimental treatment.

### Symbiont photochemical efficiency and other phenotypes

The *in situ* photochemical efficiency of the *S. ‘fitti’* strain was estimated using a Diving PAM fluorometer (Walz, Germany) with the following settings: Measuring Intensity 12, Saturation Intensity 8, Saturation Width 0.6 s, Damping 2, and Gain 3. For the cold and ambient treatments, maximum excitation pressure over photosystem II (Q_m_) was calculated daily for each colony as Q_m_ = 1 - [(ΔF/F_m_? at noon)/(F_v_/F_m_ at dusk)]^57^. Q_m_ is a normalized metric that removes the influence of daily fluctuations in solar radiation between clear and overcast days from the factor of interest (in this case temperature). It ranges from 0, where photochemistry is light-limited, to 1, indicating photoinhibition, though photosynthetic performance and pressure do not follow a linear relationship. Generally, negative effects on photosynthetic output are not observed below pressure values of 0.6 (R. Iglesias-Prieto, personal communication). To isolate the effect of cold shock, the Q_m_ of the fragment of a given colony in the ambient treatment was subtracted from the Q_m_ of the corresponding fragment of the same colony in the cold treatment to calculate ΔQ_m_. Thus, ΔQ_m_ was comparable among colonies. This normalized metric reflected the relative degree to which the clonal *S. ‘fitti’* strain adjusted photochemically to cold stress in different host genotype backgrounds.

Prior to cold exposure, each colony’s average symbiont density and cell volume were determined. Replicate hemocytometer cell counts (n = 5) were taken from a single 1 cm^2^ tissue plug per colony. Ellipsoid cell volumes were estimated as 4π(*abc*)•3^−1^, where *a* is half the cell’s longest diameter and *b* and *c* are taken as half the perpendicular diameter (n = 40 cells per colony). Additionally, all *A. palmata* colonies at the study site in Puerto Morelos, including those that were not used in this experiment, were observed for signs of annual synchronized spawning between 20:00 h and 23:00 h from Aug. 15–19 by SCUBA divers.

### Microarray experiment

The host’s acute response to cold stress was assessed through global gene expression using a microarray designed from the *A. palmata* transcriptome^58^. To incorporate biological replication, we included separate RNA extracts from the two host colonies with the smallest symbiont ΔQ_m_ (hosts *B* and *Z*) and the two host colonies with the largest symbiont ΔQ_m_ (hosts *D* and *Y*). Total RNA was extracted using the RNeasy Mini Kit (Qiagen, CA, USA) from subsamples of each host colony (n = 4) at each temperature (n = 2) taken 3.5 h after exposure to treatment. Concentration and quality of RNA extracts were quantified on a NanoDrop ND-1000 spectrophotometer (Thermo Scientific, MA, USA) and an Agilent 2100 Bioanalyzer (Agilent Technologies, CA, USA). High-quality mRNA was hybridized to custom 2-channel microarrays (Nimblegen 6019040401) following published methods^22^. Associated raw data and a more detailed description of hybridization conditions can be accessed at the NCBI Gene Expression Omnibus database through GEO Series accession number GSE50926 [http://www.ncbi.nlm.nih.gov/geo/query/acc.cgi?acc = GSE50926].

### Statistical analysis

To analyze photochemistry, ΔQ_m_ values for each fragment from all three days were included in repeated measure and one-way ANOVAs in the R statistical environment. Expression data were analyzed in R using the Bioconductor package LIMMA [http://www.bioconductor.org/packages/release/bioc/html/limma.html] following published methods^22^. A log base 2 fold change cut-off of 1.5 (=fold change cut-off of 2.8) and false discovery rate (FDR) *q*-value threshold of 0.05 were used to filter significant results. To visualize transcription profiles, expression values were transformed gene-wise into scaled coefficients (standard scores above or below the probe mean) and plotted as heat maps with MultiExperiment Viewer v4.9 [http://www.tm4.org/mev.html]. An unrooted sample tree was created through hierarchical clustering of expression profiles using the hclust function with the complete linkage agglomeration method in R. Principle component analysis was carried out using the prcomp function and a covariance matrix in R. Lists of differentially expressed genes were analyzed for functional enrichment using default parameters in the online tool GOEAST v1.3 [http://omicslab.genetics.ac.cn/GOEAST/tools.php] based on the original annotation file associated with the microarray. A list of all differentially expressed genes and the R code used in the expression analysis can be accessed in the Pennsylvania State University’s ScholarSphere database [https://scholarsphere.psu.edu/files/8623j6166].

## Additional Information

**How to cite this article**: Parkinson, J. E. *et al.* Intraspecific diversity among partners drives functional variation in coral symbioses. *Sci. Rep.* **5**, 15667; doi: 10.1038/srep15667 (2015).

## Supplementary Material

Supplementary Figures S1 and S2

Supplementary Table S1

Supplementary Table S2

Supplementary Table S3
