# Supplementary material for: Intraspecific diversity among partners drives functional variation in coral symbioses
Source: Sci Rep. 2015 Oct 26;5:15667. doi: 10.1038/srep15667 (PMC4620489; doi:10.1038/srep15667)
Supplement: Supplementary Figures S1 and S2 [file srep15667-s1.doc]

**Intraspecific diversity among partners drives functional variation in coral symbioses.**

John Everett Parkinson, Anastazia T. Banaszak, Naomi S. Altman, Todd C. LaJeunesse, Iliana B. Baums

**Supplemental Figures**

*
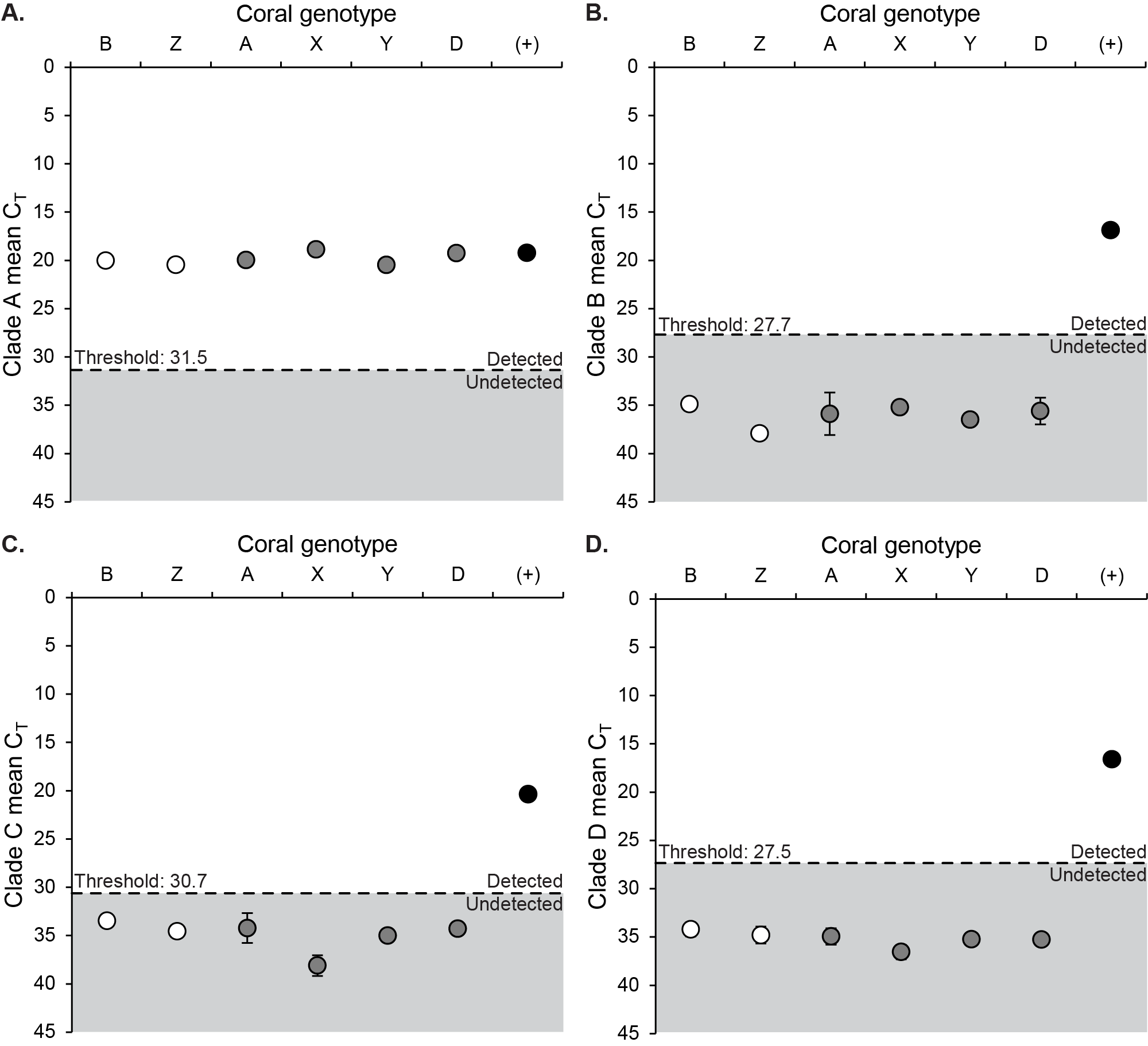
*

**Supplementary Figure S1** qPCR screening for background symbiont strains in each host colony for **(A)** Clade A, **(B)** Clade B, **(C)** Clade C, and **(D)** Clade D. Only clade A symbionts were detected. Thresholds for detection were determined by serial ten-fold dilution of DNA from monocultured *Symbiodinium* (1-0.0001 ng/µL); Clade A: rt272 (Pacific A3); Clade B: mac703 (*S. minutum*); Clade C: rt152 (*S. goreaui*); Clade D: A001 (*S. trenchii*). Each assay included a positive culture control and a no-template control. Each reaction was run in triplicate. Melting curves were used to confirm that “no detection” products were likely primer dimer (T_m_ < 80°C) and “detection” products were likely target template (T_m_ > 80°C). Dilution series analysis was conservatively restricted to C_T_ values with standard deviations within 5% of the mean across all three replicates. In addition to higher standard deviations, most excluded C_T_ values also melted at low temperature, indicating products were primer dimer. Had all dilution C_T_ values been included to extend the limit of detection of each assay, experimental C_T_ values still would have been above the threshold (indicating no detection). Error bars represent standard deviations based on three replicates. Where not visible, standard deviations were smaller than the point symbol, except for Clade B (genotypes *Z*, *X*, and *Y*) and Clade C (genotype *Z*), where only one replicate had a detectable amplification signal (and therefore no standard deviation).

**
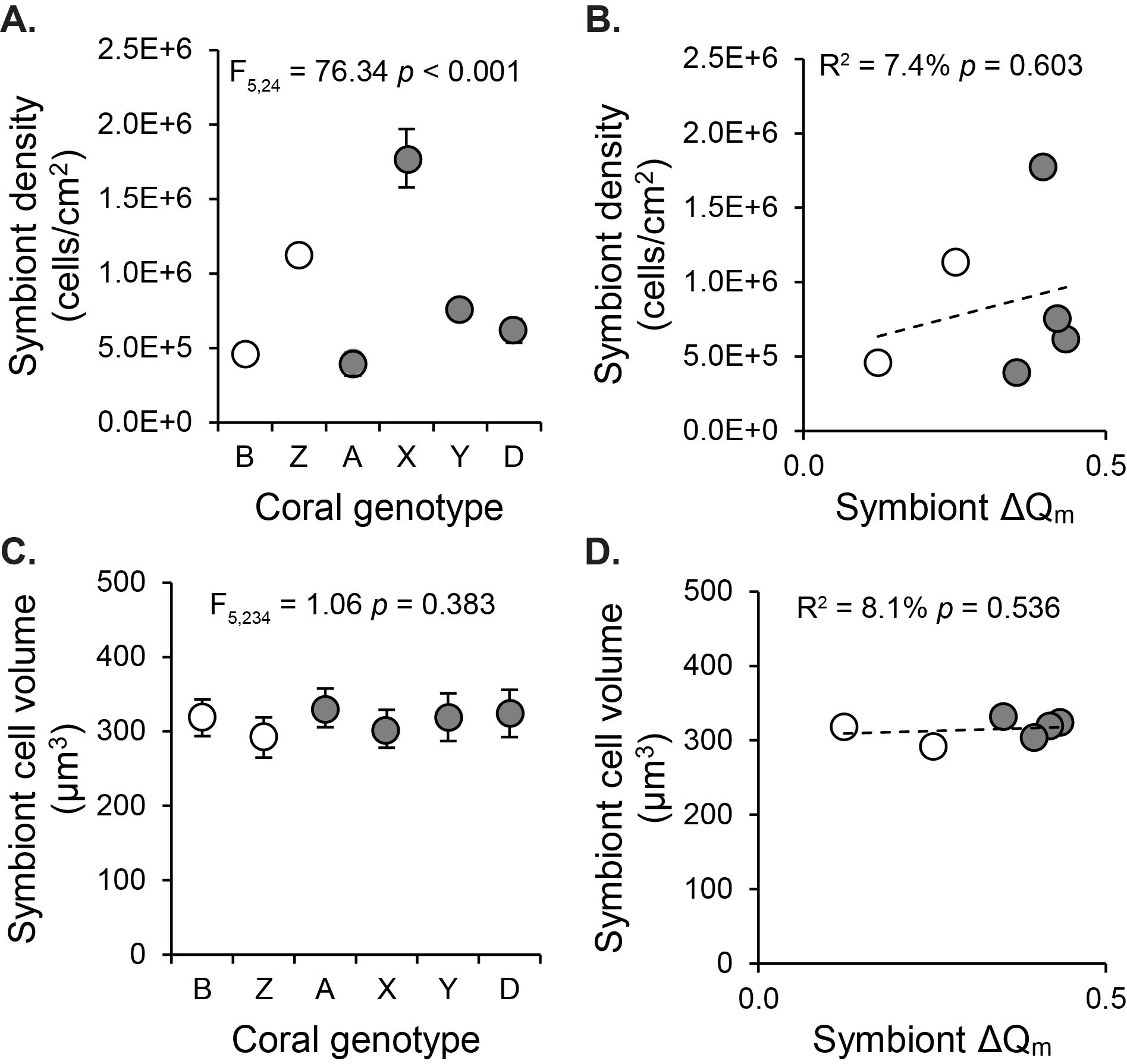
**

**Supplementary Figure S2** Phenotypes of a clonal *Symbiodinium* *‘fitti’* strain found in six genetically distinct *Acropora palmata* host backgrounds. **(A)** Average symbiont density. **(B)** Regression of symbiont density against ΔQ_m_. **(C)** Average symbiont cell volume. **(D)** Regression of symbiont cell volume against ΔQ_m_. For **(A-B)**, error bars represent 95% confidence intervals for 5 replicate hemocytometer cell counts taken from a single 1cm^2^ tissue plug per colony. To normalize for ANOVA, density was natural logarithm transformed (untransformed data plotted). For **(C-D)**, error bars represent 95% confidence intervals calculated as 4π(*abc*)•3^-1^, where *a* is half the cell’s longest diameter and *b* and *c* are taken as half the perpendicular diameter (n=40 cells per colony). To normalize for ANOVA, volume was square root transformed (untransformed data plotted). White fills indicate small ΔQ_m_ phenotypes (Dynamic colonies), while gray fills represent large ΔQ_m_ phenotypes (Static colonies).
